# The empty pelvis syndrome: a core data set from the PelvEx collaborative

**DOI:** 10.1093/bjs/znae042

**Published:** 2024-03-08

**Authors:** PelvEx Collaborative, A H Mirnezami, I Drami, T Glyn, P A Sutton, J Tiernan, C Behrenbruch, G Guerra, P S Waters, N Woodward, S Applin, S J Charles, S A Rose, A Denys, E Pape, G H van Ramshorst, D Baker, E Bignall, I Blair, P Davis, T Edwards, K Jackson, P G Leendertse, E Love-Mott, L MacKenzie, F Martens, D Meredith, S E Nettleton, M P Trotman, J J M van Hecke, A M J Weemaes, N Abecasis, E Angenete, O Aziz, N Bacalbasa, D Barton, G Baseckas, A Beggs, K Brown, P Buchwald, D Burling, E Burns, A Caycedo-Marulanda, G J Chang, P E Coyne, R S Croner, I R Daniels, Q D Denost, E Drozdov, T Eglinton, E Espín-Basany, M D Evans, K Flatmark, J Folkesson, F A Frizelle, M A Gallego, A Gil-Moreno, P Goffredo, B Griffiths, F Gwenaël, D A Harris, L H Iversen, G V Kandaswamy, M Kazi, M E Kelly, R Kokelaar, M Kusters, M C Langheinrich, T Larach, M L Lydrup, A Lyons, C Mann, F D McDermott, J R T Monson, H Neeff, I Negoi, J L Ng, M Nicolaou, G Palmer, C Parnaby, G Pellino, A C Peterson, A Quyn, A Rogers, J Rothbarth, F Abu Saadeh, A Saklani, T Sammour, R Sayyed, N J Smart, T Smith, L Sorrentino, S R Steele, K Stitzenberg, C Taylor, J Teras, M R Thanapal, E Thorgersen, W Vasquez-Jimenez, J Waller, K Weber, A Wolthuis, D C Winter, G Branagan, D Vimalachandran, A G J Aalbers, N Abdul Aziz, M Abraham-Nordling, T Akiyoshi, R Alahmadi, W Alberda, M Albert, M Andric, M Angeles, A Antoniou, J Armitage, R Auer, K K Austin, E Aytac, R P Baker, M Bali, S Baransi, B Bebington, M Bedford, B K Bednarski, G L Beets, P L Berg, C Bergzoll, S Biondo, K Boyle, L Bordeianou, E Brecelj, A B Bremers, M Brunner, A Bui, A Burgess, J W A Burger, N Campain, S Carvalhal, L Castro, W Ceelen, K K L Chan, M H Chew, A K Chok, P Chong, H K Christensen, H Clouston, D Collins, A J Colquhoun, J Constantinides, A Corr, M Coscia, M Cosimelli, C Cotsoglou, L Damjanovic, M Davies, R J Davies, C P Delaney, J H W de Wilt, C Deutsch, D Dietz, S Domingo, E J Dozois, M Duff, E Egger, J M Enrique-Navascues, E Espín-Basany, B Eyjólfsdóttir, M Fahy, N S Fearnhead, S Fichtner-Feigl, F Fleming, B Flor, K Foskett, J Funder, E García-Granero, J L García-Sabrido, M Gargiulo, V G Gava, L Gentilini, M L George, V George, P Georgiou, A Ghosh, L Ghouti, F Giner, N Ginther, T Glover, T Golda, C M Gomez, C Harris, J A W Hagemans, V Hanchanale, D P Harji, C Helbren, R M Helewa, G Hellawell, A G Heriot, D Hochman, W Hohenberger, T Holm, A Holmström, R Hompes, B Hornung, S Hurton, E Hyun, M Ito, J T Jenkins, K Jourand, S Kaffenberger, S Kapur, Y Kanemitsu, M Kaufman, S R Kelley, D S Keller, S Kersting, S H J Ketelaers, M S Khan, J Khaw, H Kim, H J Kim, R Kiran, C E Koh, N F M Kok, C Kontovounisios, F Kose, M Koutra, M Kraft, H Ø Kristensen, S Kumar, V Lago, Z Lakkis, B Lampe, S G Larsen, D W Larson, W L Law, S Laurberg, P J Lee, M Limbert, A Loria, A C Lynch, M Mackintosh, C Mantyh, K L Mathis, C F S Margues, A Martinez, A Martling, W J H J Meijerink, A Merchea, S Merkel, A M Mehta, D R McArthur, J J McCormick, J S McGrath, A McPhee, J Maciel, S Malde, S Manfredelli, S Mikalauskas, D Modest, J R Morton, T G Mullaney, A S Navarro, J W M Neto, B Nguyen, M B Nielsen, G A P Nieuwenhuijzen, P J Nilsson, S Nordkamp, S T O’Dwyer, K Paarnio, E Pappou, J Park, D Patsouras, O Peacock, F Pfeffer, F Piqeur, J Pinson, G Poggioli, D Proud, M Quinn, A Oliver, R W Radwan, N Rajendran, C Rao, S Rasheed, P C Rasmussen, E Rausa, S E Regenbogen, H M Reims, A Renehan, J Rintala, R Rocha, M Rochester, J Rohila, M Rottoli, C Roxburgh, H J T Rutten, B Safar, P M Sagar, A Sahai, A M P Schizas, E Schwarzkopf, D Scripcariu, V Scripcariu, G Seifert, C Selvasekar, M Shaban, I Shaikh, D Shida, A Simpson, T Skeie-Jensen, P Smart, J J Smith, A M Solbakken, M J Solomon, M M Sørensen, M Spasojevic, D Steffens, L Stocchi, N A Stylianides, T Swartling, H Sumrien, T Swartking, H Takala, E J Tan, D Taylor, P Tejedor, A Tekin, P P Tekkis, H V Thaysen, R Thurairaja, E L Toh, P Tsarkov, J Tolenaar, Y Tsukada, S Tsukamoto, J J Tuech, G Turner, W H Turner, J B Tuynman, M Valente, J van Rees, D van Zoggel, W Vásquez-Jiménez, C Verhoef, M Vierimaa, G Vizzielli, E L K Voogt, K Uehara, C Wakeman, S Warrier, H H Wasmuth, M R Weiser, O L Westney, J M D Wheeler, J Wild, M Wilson, H Yano, B Yip, J Yip, R N Yoo, M A Zappa

## Abstract

**Background:**

Empty pelvis syndrome (EPS) is a significant source of morbidity following pelvic exenteration (PE), but is undefined. EPS outcome reporting and descriptors of radicality of PE are inconsistent; therefore, the best approaches for prevention are unknown. To facilitate future research into EPS, the aim of this study is to define a measurable core outcome set, core descriptor set and written definition for EPS. Consensus on strategies to mitigate EPS was also explored.

**Method:**

Three-stage consensus methodology was used: longlisting with systematic review, healthcare professional event, patient engagement, and Delphi-piloting; shortlisting with two rounds of modified Delphi; and a confirmatory stage using a modified nominal group technique. This included a selection of measurement instruments, and iterative generation of a written EPS definition.

**Results:**

One hundred and three and 119 participants took part in the modified Delphi and consensus meetings, respectively. This encompassed international patient and healthcare professional representation with multidisciplinary input. Seventy statements were longlisted, seven core outcomes (bowel obstruction, enteroperineal fistula, chronic perineal sinus, infected pelvic collection, bowel obstruction, morbidity from reconstruction, re-intervention, and quality of life), and four core descriptors (magnitude of surgery, radiotherapy-induced damage, methods of reconstruction, and changes in volume of pelvic dead space) reached consensus—where applicable, measurement of these outcomes and descriptors was defined. A written definition for EPS was agreed.

**Conclusions:**

EPS is an area of unmet research and clinical need. This study provides an agreed definition and core data set for EPS to facilitate further research.

## Introduction

The empty pelvis syndrome (EPS) is a poorly understood set of related complications that occur after pelvic exenteration (PE). Despite the lack of an agreed definition, it is recognized as a significant cause of morbidity in up to 40% of patients following PE, causing pelvic sepsis and perineal complications^1^. EPS was first described in 1993: ‘The empty pelvis syndrome, in the early phase, may resemble a flulike illness with malaise, elevated temperature, and increased discharge from the perineal sinus that may continue for many years, particularly among those undergoing heavy irradiation.’^2^ The pathophysiology responsible for EPS is largely unknown, and is likely to interact with other complications of PE, such as those relating to perineal wounds or urinary reconstruction.

Through surgical advances more radical PEs are frequently now performed. Patients who undergo extended PE, with major bone or nerve resection, may require significantly more interventions to manage complications of EPS^3,4^. The prevalence of EPS is an area of increased research interest, with five references from 1993 to 2014^2,5–8^ and 24 between 2015 and 2022 (summarized in *Table S1*)^1,3,9–30^. The definitions of EPS within this literature are inconsistent, with heterogeneity in the reported contributory pathophysiological factors and outcomes. This has confounded data synthesis, with a systematic review assessing reconstructive techniques to mitigate EPS unable to draw strong conclusions in favour of a particular strategy, resulting in ‘research waste’ and heterogeneous clinical practice^1^.

A core outcome set (COS) is an agreed standardized collection of outcomes that should be measured and reported, as a minimum, in trials on a specific area of health^31^. A core descriptor set (CDS) defines the minimum patient characteristics that should be reported in future research, again with the purpose of reducing such heterogeneity^32^. To avoid persistent heterogeneity following agreement of a COS and a CDS, it should then be determined how these are defined and measured^31^. Developing consistent outcome reporting and patient characteristic descriptors will address current research challenges, facilitating future study design, meta-analysis, and advancement of the field in order to reduce the morbidity around EPS.

Techniques to mitigate EPS involve filling or excluding the pelvic dead space created after PE. These include meshes, breast prostheses, myocutaneous flaps, omentoplasty, obstetric balloons, silicone tissue expanders, inflated Foley catheters, lipofilling, and mobilization of nearby peritoneum^1,9,19,33^. Exploring consensus on these strategies will assist in establishing best practice and identifying research priorities.

This project was performed as part of the PelvEx Collaborative (PelvEx), an international group of healthcare professionals providing PE surgery from over 140 units across five continents. The aim of this study is to facilitate the design of future research on EPS with three objectives:

1. Generate a measurable EPS core outcome set.
2. Establish consensus on EPS pathophysiology to generate a measurable core descriptor set and written definition.
3. Explore consensus on strategies to mitigate EPS.

## Methods

This study used Guidance on Conducting and REporting DElphi Studies (CREDES), the Core Outcome Measures in Effectiveness Trials (COMET) handbook, Consensus-based Standards for the selection of health Measurement Instruments (COSMIN)/COMET guidance, and Core Outcome Set-STAndards for Reporting (COS-STAR) Guidelines^31,34–36^. A three-stage design was used: first, longlisting statements through systematic review, a healthcare professional event, patient and public involvement (PPI), and Delphi-piloting; second, shortlisting statements using two rounds of online modified Delphi; and finally confirming statements and agreeing measurements with virtual patient-representative consensus meetings, and a face-to-face healthcare professional consensus meeting. The study was added to the COMET database^37^, and a protocol registered on ClinicalTrials.gov (NCT05683795) prior to the shortlisting stage.

### Stage 1—longlisting statements

Statements were divided into three domains to meet study aims:

1. EPS core outcome set.
2. EPS pathophysiology.
3. EPS mitigation.

A systematic review and subsequently published literature on EPS were searched for using statements matching these domains^1,5,28^. An EPS initiative was presented at PelvEx 2022 in Amsterdam where further statements were generated based on formal and informal discussion. A study steering committee was formed from an international group of healthcare professionals, patient representatives that had undergone PE, and PPI professionals from the charity Bowel Research UK (BRUK). Further statements were developed from discussions and Delphi-piloting within this group.

### Stage 2—modified Delphi shortlisting statements

An online modified Delphi was undertaken. There is no agreement on how groups of experts should be selected in a Delphi, and they are not required to be statistically representative^38^. All members of PelvEx were invited to take part to form a healthcare professional group. Patient representatives were also recruited—inclusion criteria being any individual that had undergone PE, defined as oncological resection of multiple pelvic organs, including beyond total mesorectal excision (TME) operations. Translation for non-English speakers was provided from appropriate multilingual members of PelvEx, and LanguageInsight. Patient advocacy groups BRUK, World Federation of Incontinence and Pelvic Problems, and CommunitiesFirst facilitated identification and recruitment of patient representatives; and members of PelvEx were encouraged to engage their individual institutional PPI networks. Demographic information was collected to report the diversity of stakeholder groups.

Qualtrics^TM^ was used to run the online modified Delphi using the ‘force response’ function to minimize incomplete data. This was initially piloted with 15 participants to refine the platform prior to wider dissemination. Longlisted statements were displayed by domain in alphabetic order to minimize leading questions or researcher bias. Patient representatives were only invited to participate in the COS domain; therefore, these statements were presented in lay terms with technical language in parentheses. This decision was made following discussions with patient representatives on the steering committee, who did not feel able to contribute helpful consensus to the pathophysiology and mitigation domains.

Participants scored statements from 1 to 9 on a Likert scale, as recommended by the Grading of Recommendations Assessment, Development and Evaluation (GRADE) working group^39^. Scores of 1–3 represented ‘not important’, 4–6 represented ‘important but not critical’ and 7–9 represented ‘critical for inclusion.’ A score of ‘0’ was also included, meaning ‘unable to comment’. In the first round, final questions of each domain were open to avoid early closure of ideas and generate new statements. In addition, in the mitigation domain, open questions were used to survey current reconstructive practice. Open questions were not mandatory and were not used in later rounds.

There is no recognized method to define consensus in Delphi studies. For the present study, it was specified *a priori* that for statements to progress from the first round they had to be rated 7–9 by 50% or more of participants and by 1–3 by no more than 15% of participants in at least one stakeholder group. To progress from the second round, statements had to be rated between 7 and 9 by over 70% of participants and by 1–3 by less than 15% of participants by at least one stakeholder group. This approach reduced exclusion of statements potentially rated more highly in subsequent rounds once participants had received feedback. During piloting a high proportion of statements in the COS domain were rated as ‘critical for inclusion’; therefore, it was specified *a priori* that if there were 10 or more COS statements reaching consensus by the end of the second round, then a third round would take place with higher levels of consensus required, defined as 95% of participants voting a statement as 7–9. This approach was based on previous COS studies^40,41^.

To minimize attrition the ‘mobile-friendly’ function on Qualtrics^TM^ was used. During piloting the average time taken for completion was calculated and communicated on participant information sheets, and personalized reminders were sent to participants 2 weeks and 48 hours before rounds closed. Each round remained open for one month. Attrition between rounds was determined; if dropping below 70%, the modified Delphi stage of the study would be terminated, as rigour could not be guaranteed. Participants were encouraged to give reasons for discontinuation to support attrition analysis. Attrition bias was assessed by calculating average scores across all statements from all domains for each participant within each stakeholder group. A comparison was made between individuals that completed round one only against those that completed both rounds one and two^42^.

Responses to open questions from the first round underwent thematic analysis by the study steering committee and were used to merge, refine or formulate new statements for subsequent rounds. Comments applying to already longlisted statements or domains were presented in relevant sections in subsequent rounds. Medians and interquartile ranges were used for quantitative analysis, which was undertaken separately for patient representatives and healthcare professionals. It was anticipated that more healthcare professionals would participate, and this approach was designed to prevent reduction of the importance given to the patient voice. Histograms were produced to facilitate understanding for those unfamiliar with descriptive statistics. Individuals were sent bespoke feedback on how they voted in previous rounds using Microsoft Office Mail Merge. Data analysis was performed in Microsoft Excel, OpenRefine and R Studio.

### Stage 3—consensus meetings for confirming statements and agreeing measurements

Separate patient representative and healthcare professional consensus meetings were held using a modified nominal group technique to discuss and confirm whether shortlisted statements should appear in the final domains, and to agree how the COS and CDS should be measured^43^. The mitigation domain was not voted upon at this stage.

Prior to consensus meetings, shortlisted statements that reached consensus by the end of the modified Delphi had options for instruments prepared in accordance with the COSMIN/COMET four-step guideline^35^. To generate a CDS, statements from the pathophysiology domain that were measurable also had instruments prepared in the same manner. Conceptual considerations were considered completed by the modified Delphi process. For binary statements, whose measurement is already well established, COSMIN principles were not applicable^44^. In these cases, precise definitions were sought to enable consistent measurement and controversies identified for presentation at consensus meetings. Systematic reviews on PE, relevant MEDLINE and EMBASE searches and input of the steering committee were used to identify existing measurement instruments. Quality assessment and feasibility aspects for patient-reported outcome measures (PROMs) and clinician-reported outcome measures (ClinROMs) were undertaken using relevant COSMIN risk of bias assessments^45,46^. Finally, instruments were selected by voting at consensus meetings; if no suitable instrument existed, then a recommendation for further validation work could be made.

It was not feasible to invite patient representatives to a face-to-face consensus meeting, and time zone differences prevented organization of a single virtual meeting. Patient representatives were therefore invited to four virtual consensus meetings over Microsoft Teams. These were supported by an independent PPI professional and an appropriate translator from PelvEx institutions. Votes were taken on shortlisted statements for inclusion into the final COS and aggregated across meetings. Instruments prepared for the COS were presented for approval, with patients judging relevance, comprehensiveness, and comprehensibility. Shortlisted statements in the pathophysiology domain were also presented for feedback. Patient representatives consented to video recordings describing their personal experiences of adverse EPS outcomes.

The face-to-face healthcare professional consensus meeting took place at PelvEx 2023 in Bordeaux with 90 min on the academic programme. Prior to this, results from the second Delphi round, virtual patient consensus meetings and questions for the consensus meeting were disseminated in advance to all delegates using a quick response (QR) code. A list of delegates attending the final consensus meeting was obtained, and it was established whether they had taken part in the Delphi process. In lieu of patient representatives being able to attend, the video recording from the patient consensus meetings was shown to communicate how shortlisted outcomes impacted on patients. An audio recording of the meeting was made to facilitate analysis. Mentimeter live voting software was used to anonymously enable the modified nominal group technique, producing histograms in real time. Participants voted in a binary manner, with the option of being unsure also available. Where there was no clear agreement, further discussion was encouraged to capture dissenting views to determine the nature of the polarized response. Further voting took place, and if there was persistent disagreement further discussion was followed by a final vote using majority rule. Participants were asked to vote on the final inclusion of statements for the COS and pathophysiology domains. Immediately following this, pre-prepared instruments for the COS and CDS were discussed and voted upon in the same manner.

To systematically generate a written definition of EPS, all shortlisted pathophysiology statements were combined to formulate a written definition. This was iteratively discussed and edited in live time. Further online voting was protocolized if not all study aims were achieved. Deviation from the presented protocol and decisions on stoppage or continuation of the modified Delphi were undertaken by the study steering committee.

### Research ethics

This study was approved by the University of Southampton Faculty of Medicine ethics board (ERGO II reference number 77306), Te Whatu Ora Health New Zealand (RO# 23020) and the ethics board of Ghent University Hospital in Ghent, Belgium (ONZ-2023-0099). An online informed consent form was displayed when accessing the Delphi Qualtrics^TM^ link; the ‘force response’ function was used to ensure consent was obtained before participants could access the study.

### Patient and public involvement

Patient representatives with lived experience of PE were recruited onto the study steering committee and embedded into the project from the outset through co-design of the methodology. Further patient engagement was facilitated through BRUK, World Federation of Incontinence and Pelvic Problems, and CommunitiesFirst. PelvEx members were encouraged to engage their own PPI networks.

### External validation

Prior to submission the project was independently approved by the Association of Coloproctology of Great Britain and Ireland, Research and Audit committee.

## Results

### Stage 1—longlisting statements

**Fig. 1:**
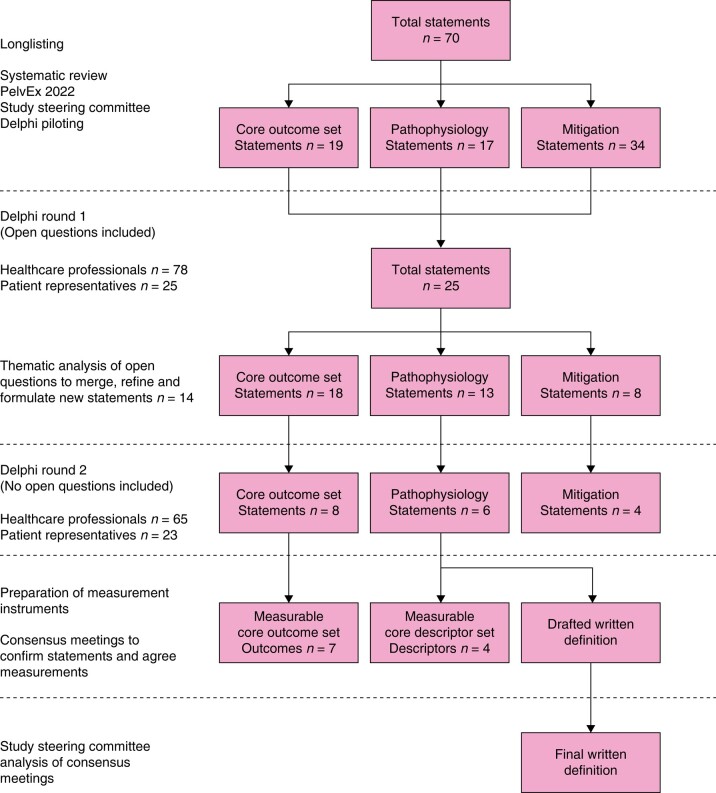
Study flow diagram showing longlisting, shortlisting and confirmatory stages

A longlist of 70 statements was produced for the first Delphi round, displayed in *Table S2*. The number of statements for each domain were: EPS COS, 19 statements; EPS pathophysiology, 17 statements; and EPS mitigation, 34 statements. Results are summarized in *Fig. 1*.

### Stage 2—modified Delphi shortlisting

In the modified Delphi 78 healthcare professionals and 25 patient representatives participated, whose characteristics are summarized in *Tables 1*, *2*. There were 24 different nationalities within the healthcare professional group, multidisciplinary input was from six specialities, with 1623 cumulative years in clinical practice. Patient representatives were from four nations, 56% (14/25) reported a total PE, 64% (16/25) reported infralevator resection, 14/25 (56%) reported flap reconstructions, and 5/25 (20%) reported surgical mesh reconstruction.

**Table 1:** Demographics of healthcare professionals participating in the modified Delphi

	Healthcare professionals (*N* = 78)
**Sex**	
Male	31
Female	8
**Age**	
Median (i.q.r.)	47 (11)
**Clinical seniority (years)**	
Median (i.q.r.)	20 (9.8)
**Profession (%)**	
Cancer nurse specialist	1 (1.3)
Colorectal surgeon	65 (83.3)
Gynaecology surgeon	5 (6.4)
Radiologist	1 (1.3)
Surgical oncologist	2 (2.6)
Urologist	4 (5.1)
**Country of residence (%)**	
Australia	4 (5.1)
Belgium	1 (1.3)
Canada	1 (1.3)
Chile	1 (1.3)
Denmark	1 (1.3)
Estonia	1 (1.3)
France	2 (2.6)
Germany	4 (5.1)
India	2 (2.6)
Ireland	4 (5.1)
Italy	1 (1.3)
Malaysia	1 (1.3)
Netherlands	3 (3.8)
New Zealand	3 (3.8)
Norway	2 (2.6)
Pakistan	1 (1.3)
Portugal	1 (1.3)
Romania	2 (2.6)
Russia	1 (1.3)
Singapore	1 (1.3)
Spain	5 (6.4)
Sweden	6 (7.7)
UK	23 (29.5)
USA	7 (9.0)

**Table 2:** Demographics of patient representatives participating in the modified Delphi

	Patient representatives (*N* = 25)
**Sex**	
Male	9
Female	16
**Age**	
Median (i.q.r.)	55 (20)
**Years post-surgery (%)**	
Median (i.q.r.)	2.0 (3.3)
Missing	1 (4.0)
**Patient-reported pelvic exenteration (%)** ^47^	
Did not know	1 (4.0)
Infralevator posterior pelvic exenteration	6 (24.0)
Infralevator total pelvic exenteration	10 (40.0)
Supralevator anterior pelvic exenteration	1 (4.0)
Supralevator posterior pelvic exenteration	3 (12.0)
Supralevator total pelvic exenteration	4 (16.0)
**Patient-reported reconstruction (%)**	
Did not know	4 (16.0)
Gluteal flap	7 (28.0)
No reconstruction	2 (8.0)
Rectus flap	2 (8.0)
Rectus flap and omentoplasty	1 (4.0)
Surgical mesh	5 (20.0)
Thigh flap	2 (8.0)
Thigh flap and gluteal flap	2 (8.0)
**Country of residence (%)**	
Australia	1 (4.0)
Canada	2 (8.0)
New Zealand	4 (16.0)
UK	18 (72.0)

Across all three domains 25/70 statements proceeded into the second round, and thematic analysis from open questions generated 14 new statements, giving a total of 39 statements in the second round (*Table S3*). The second round was completed by 88/103 (85.4%) of all participants, 65/78 (83.3%) of healthcare professionals, and 23/25 (92%) of patient representatives. Individuals that completed round one only (*n* = 15) were compared against those completing both rounds one and two (*n* = 88). This demonstrated no extreme views in those failing to complete round two, suggesting attrition bias had not occurred between rounds—see *Fig. S1*. Following the second round, 18/39 statements reached consensus, with less than 10 COS statements remaining; therefore, a third Delphi round was not required.

The four shortlisted statements from the mitigation domain are summarized in *Table 3*. Responses to open questions on preventative strategies from the first Delphi round were divided into use of flaps, prostheses and surgical drains. The highest frequency flaps reported were rectus 29/70 (41.4%), gluteal 14/70 (20%) and gracilis flaps 9/70 (12.9%). More meshes were reported as collagen tissue matrices 10/16 (62.5%), with 4/16 (25%) using absorbable bioprosthetic mesh. Operatively placed drains were of variable types, position and time left *in situ* prior to removal—further detail is given in *Table S4*. Mitigation statements that reached a high level of disagreement in the first Delphi round are shown in *Table 4*.

**Table 3:** Shortlisted statements in the empty pelvis syndrome mitigation domain, class of recommendation and level of evidence of clinical guidelines are given

Empty pelvis syndrome mitigation domain statements reaching consensus	Voted by >70% as ‘7–9’, critical for inclusion (%)	Strength of recommendation
Mobilization of structures to fill or prevent small bowel migrating to the pelvis that is bladder, uterus, caecum, small bowel mesentery	73.8	Class IIA/Level C
Bulky myocutaneous flaps to fill pelvis and achieve perineal coverage	89.2	Class I/Level C
Omentoplasty to fill pelvis	80.0	Class I/Level C
Multiple techniques to fill the pelvis	87.7	Class I/Level C

Class I denotes weight of consensus opinion is in favour of efficacy. Level C demonstrates evidence based on expert opinion, small studies, and retrospective studies only^48,49^.

**Table 4:** Strategies to mitigate the impact of EPS that were not viewed as strongly in this study

Empty pelvis syndrome mitigation domain statements reaching consensus	Voted ‘1—3’ as not important (%)
Saline-filled breast prosthesis to fill the pelvis	50.0
A silicone breast prosthesis to fill the pelvis	54.1
No deliberate manoeuvres used to fill the pelvis	54.1
Plication of a loop of small bowel onto the pelvic brim to prevent other small bowel loops falling into the pelvis	60.8
The continued prophylactic use of postoperative antibiotics to prevent infected fluid collections	55.4
Non-absorbable synthetic mesh	58.1
Multiple Foley catheters placed into the pelvis and deflated over time	59.5
Use of deliberate techniques to encourage the small bowel to form adhesions so it does not fall into the pelvis	60.8

### Stage 3—consensus meetings for confirming statements and agreeing measurements

In the COS and pathophysiology domains 14 statements reached the confirmation stage. Virtual patient representative meetings were attended by 12 individuals, 8/12 (66.7%) participated in the shortlisting process, 8/12 (66.7%) were male, 4/12 (66.7%) were female, 8/12 (66.7%) resided in the UK, 3/12 (25%) in the Netherlands and 1/12 (8.3%) in Belgium. Eight of 12 (66.7%) spoke English and 4/12 (33.3%) Dutch/Flemish. Within this unselected cohort, patients experienced 10 of the shortlisted outcomes: one bowel obstruction, two infected pelvic collections, two pelvic interventional radiology drains, four chronic perineal sinuses and one flap infection. Patient representatives described experiences of these adverse outcomes and salient points were shown at the healthcare professional meeting. Shortlisted pathophysiology statements and proposals for measuring the COS and CDS were presented.

The face-to-face healthcare professional consensus meeting had 117 attendees, 87/117 (74.4%) males, 30/117 (25.6%) females, nine professional groups were represented including colorectal, surgical oncology, radiology, plastics, urology, gynaecology, radiation oncology and vascular. Nationalities from 24 countries across five continents were represented. One hundred and seven of 117 (91.5%) delegates took part in the online live Mentimeter voting, of whom 46/107 (43.0%) took part in the modified Delphi process. Voting on statements for inclusion into the final EPS COS and EPS pathophysiology domain is summarized in *Table S5*.

Prior to meetings consensus questions for voting on measuring the shortlisted COS and CDS statements were prepared. COSMIN preparatory work to justify these questions is described in the Appendix. Note that preparatory work for postoperative mortality was overlooked at the consensus meeting, as it was excluded in the first session of voting. The final measurable COS and CDS are shown in *Table 5*.

**Table 5:** Summary of the final measurable empty pelvis syndrome core outcome set and core descriptor set

A measurable core outcome set for the empty pelvis syndrome	
Outcome	Measurement
Bowel obstruction	On CT demonstrating a transition point in the pelvis, with no time constraint.
Enteroperineal fistula	Any connection between small bowel or colon and the perineal wound or pelvic viscera to drain through the perineum. With no time constraint.
Chronic perineal sinus	Chronic fluid discharging through an unhealed perineal wound or through remnants of pelvic viscera present at least 6 months following surgery.
Infected pelvic fluid collection and pelvic abscess	On a CT reported by a radiologist, describing an infected pelvic fluid collection or pelvic abscess, with no time constraint. This includes any infected collection within the neo-perineum, defined as a collection inferior to any reconstruction of the pelvic floor. To include infected pelvic collections in patients with either intestinal or urinary anastomotic leaks; however, presence of any anastomotic failure should be reported.
Return to theatre and use of interventional radiology for EPS complications	Empty pelvis syndrome complications should be scored using the Clavien–Dindo classification, the reason for any re-intervention, and the re-intervention performed should be stated.
Morbidity from reconstruction	Morbidity from any strategy to reconstruct the empty pelvis should be given. In cases of flaps this should include: Major flap dehiscence (requiring secondary surgical closure) Minor flap dehiscence (not requiring surgical closure, but including those requiring vacuum dressing) Flap necrosis (requiring operative debridement) Partial flap loss (requiring dressings, bedside debridement or vacuum dressing) Fluid collection (haematoma or seroma requiring drainage) Local infection (requiring antibiotics only) Donor site morbidity In cases of implants used for reconstruction this should include: Device failure Implant removal
Quality of life	Postoperative quality of life should be recorded; however, ongoing validation work is required before a single instrument can be recommended to consistently evaluate this.

A measurable core descriptor set for the empty pelvis syndrome	
Descriptor	Measurement
Radiotherapy-induced damage	Dosages of preoperative radiotherapy should be reported in Gy; this should be cumulative if re-irradiation is given. Different modalities of radiotherapy (external beam radiotherapy, intraoperative electron radiotherapy or intraoperative brachytherapy) should be reported in Gy separately.
Magnitude of surgery	Radicality of resection should be reported using the pelvic exenteration lexicon.^50^
Methods of reconstruction	The detail on any strategy used to fill and reconstruct the empty pelvis should be given.
Changes in the volume of pelvic dead space	Further validation work is required to evaluate whether volumetric changes in pelvic dead space reliably correlate to complications of EPS.

Statements that were voted into the final pathophysiology domain were combined to generate a written definition of EPS. This was iteratively modified at the consensus meeting, but it was not possible to precisely reach consensus with a large multinational audience. The study steering committee then analysed the audio recording from the face-to-face meeting and agreed a written definition of EPS:

The empty pelvis syndrome encompasses a spectrum of post-exenteration complications including infected fluid collections, bowel obstruction, perineal sinus, and fistulas—severity is multifactorial, likely due to radicality of resection and migration of bowel into the void generated.

## Discussion

EPS is a challenging problem faced by both patients and clinicians, causing substantial morbidity after PE. The best approach to prevent it is unknown and surgical practice varies. The current literature is confounded by inconsistent definitions, uncertainty surrounding contributing factors and pathophysiology, and heterogeneous outcome reporting. This study therefore aimed to reach consensus on a measurable COS, measurable CDS and written definition for EPS, and to explore consensus on current strategies used to mitigate EPS.

This has been achieved with a diverse international group of healthcare professionals with multidisciplinary input from colorectal, gynaecological, surgical oncology, plastic surgery, radiology, radiation oncology and clinical nurse specialists. In addition, patient representatives have been involved throughout, in study co-design, steering committee involvement, shortlisting and confirmatory stages. Patient representatives were diverse, with multinational representation and a range of both lived experiences in radicality of PE resection and reconstruction.

In the absence of previous clear definitions, high quality data, and pre-existing consensus, a modified Delphi approach was utilized to converge and collate collective intelligence and to help focus and facilitate future higher quality research. A critique of a modified Delphi approach is that the initial selection of statements by the steering committee can introduce bias; however, in this study, open questions in the first round allowed for further suggestions, with 3/17 of the final statements being generated in this manner^51^. This approach therefore encouraged new ideas, while also grounding the study in previously developed work.

### Core outcome set

EPS is likely to be a spectrum of morbidity, with less-severe complications that can be conservatively managed. These include non-infected pelvic fluid collections, prolonged ileus and perineal wound infections—none of which reached consensus in this study. In some and particularly after high-complexity PE^3^, the complications above are more likely to progress to pelvic abscesses, small bowel obstruction and chronic perineal sinus formation, respectively. The defined COS in this study does not cover this spectrum in its totality; however, enteroperineal fistula, infected pelvic fluid collection, chronic perineal sinus and bowel obstruction in the pelvis represent the more severe sequelae of EPS. An important consideration in the development of infected pelvic fluid collections are those caused by anastomotic leakage, particularly when such a leak occurs from a join that has fallen down into the empty pelvic cavity. It is challenging to establish whether this is completely due to EPS, if EPS contributed or whether the leak was a distinct problem with the anastomosis itself. Rather than excluding such complications from the COS it has been stipulated that co-existence of leaks with an infected pelvic fluid collection should be specified.

Each of the above complications can be variable in their severity, some being manageable conservatively and others requiring re-intervention. Collecting data on re-interventions will assist in delineating severity of morbidity from EPS, as well as giving further insight into management strategies for EPS complications when they occur. In the EPS mitigation domain flaps were the only techniques to reach consensus, which included the use of multiple techniques to fill the pelvis. If this strategy was to incorporate multiple flaps, then although morbidity from the EPS itself could be reduced, donor site morbidity could be considerable. Conversely surgical devices may only be prone to failure or removal. Inclusion of morbidity from EPS reconstruction in the COS therefore will support identification of the safest reconstructive techniques.

The outcome, quality of life, is broad, and a classical Delphi engaging patient representatives qualitatively in the first round could have added additional insight into how EPS affects health-related quality of life. At that time, however, there was no agreed EPS written definition, the COS had not been delineated, and it was felt beyond the scope of the current study. Complications following PE should be considered in any specific PROM in this population and in lieu of a modified Delphi, undertaking qualitative interviews with patients that have suffered EPS would address this shortcoming.

### Core descriptor set

The morbidity experienced from EPS will not only be reliant on the type of reconstruction performed to mitigate its effects, but is likely to be a reflection of the extent of surgical resection and neoadjuvant treatments that a patient has had. Focusing only on outcomes for future research on EPS could therefore give misleading conclusions. A patient undergoing a beyond-TME extra-levator abdominoperineal excision with just lymphadenectomy of involved sidewall nodes is likely to have a different level of morbidity risk from EPS when compared to an individual undergoing total infralevator PE with sacrectomy. By specifying a CDS, the factors considered most important will be captured. Until recently, defining the extent of resection following PE was challenging (see Appendix). Utilization of the PE lexicon facilitates standardized reporting, in particular delineating differences in morbidity between high-complexity PE and conventional PE^50^. There is consensus that bowel falling into the empty pelvic cavity is an important contributor to EPS, with most reconstructive techniques designed to prevent this occurring. A loop of small bowel incarcerated deep into the pelvis is more likely to become obstructed, fistulate or allow bacterial translocation to occur across its lumen, leading to infection of postoperative fluid collections and pelvic sepsis. Radiotherapy has been implicated in previous papers as contributing to EPS, and also reached consensus^2,3,8,9,13,15,18,23,24^. This is likely to be multifactorial, with radiation-induced fibrosis leading to relative ischaemia within an empty pelvic cavity leading to reduced resistance to septic complications; in addition, radiation enteritis of bowel loops falling into the empty pelvis are more likely to cause EPS complications.

**Fig. 2:**
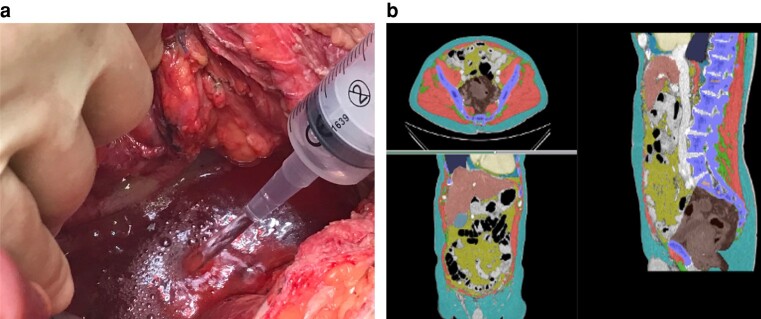
**a**, Resectional phase of pelvic exenteration is completed and the perineum reconstructed so that it is water-tight; to establish the volume of increased pelvic dead space, the table is tilted into the reverse-Trendelenburg position so the fluid level is parallel to the pelvic inlet (between sacral promontory and pubic symphysis) and the volume of saline required to fill this space is recorded. **b**, Preoperative scan using Data Analysis Facilitation Suite (DAFS) version 3.6 by Voronoi, deep learning-driven software capable of automated CT scan 3D segmentation courtesy of the BiCyCLE group at St Mark's Hospital; this may predict the increased volume of pelvic dead space based on surgical roadmap planning and the line of the pelvic inlet—the area in brown is the planned resectional specimen

Loss of domain in the context of massive ventral hernias has undergone systematic review and definition with an international Delphi consensus^52,53^. In loss of domain there are also pathological changes in the volume of the peritoneal cavity, and as well as a written definition, a volumetric definition has been achieved using the Sabbagh method^54^. Given the uncertainty of EPS, pursuit of a volumetric definition to enable objective assessment is attractive; this is reflected in the CDS with ‘changes in volume of pelvic dead space’. Crude conceptual work is underway to develop a volumetric definition for EPS using ratios of change in pelvic dead space before and after PE, which may be replicable with 3D pelvimetry CT segmentation using preoperative imaging, see *Fig. 2*^55,56^.

Establishing the relationship between EPS-related morbidity and volumetric changes may facilitate objective quantification of preoperative pelvic volume to assist in planning of reconstruction to replace lost pelvic volume. This hypothesis, however, relies only on the consensus assumption that changes in volume of dead space are important.

### Strategies to mitigate the empty pelvis syndrome

Mitigation strategies reaching consensus were omentoplasty, bulky myocutaneous flaps, mobilization of other structures, and use of multiple techniques for pelvic filling. The evidence to support these strategies is of low level, and reliant upon the expert opinion given here, or on largely retrospective studies. Prosthetic devices of any type did not reach consensus, despite favourable outcomes published using biosynthetic Bio-A® (GORE®) mesh and Bakri obstetric balloons^26,27^. The mitigation domain had the largest number of longlisted statements and smallest number of shortlisted statements, which likely reflects the high variability in reconstructive techniques currently utilized, with no single method being widely adopted.

### Strengths and limitations

It was not possible to invite patient representatives to the face-to-face meeting; however, this was managed by showing a video of how EPS had affected patient representatives. At the healthcare professional consensus meeting the majority of delegates had not taken part in the Delphi process. This was anticipated and managed by circulation of the QR code, giving a report on the study and the questions asked at the consensus meeting. This combined with use of Mentimeter live voting software then enabled 39 questions to be efficiently posed, and for consensus to be reached on all of them in the time available. The modified Delphi process was validated by this wider audience, with only one shortlisted statement in the COS and pathophysiology domains failing to reach final consensus.

This study has generated a measurable COS, measurable CDS and written definition for EPS. This reduces the need for further consensus studies in a clear area of unmet research and clinical need. This consensus should now be tested in feasibility studies prior to larger-scale international observational work. This will enable a better understanding of the effects that radicality of PE, radiotherapy and reconstructive techniques have on adverse EPS outcomes. A Delphi study is a heuristic device that relies on expert knowledge to co-construct knowledge and recommendations. It is only as good as the available evidence and the participating experts. The available evidence on EPS is known to be of poor quality, and this study does not produce any new evidence on EPS. Furthermore, EPS is likely to interact with many pathological processes that occur after PE, and there is a risk of attributing all complications following PE to EPS by placing several diagnoses under the name of one disease. Findings reported here should therefore be followed with caution and in light of larger volumes of more consistent data in the future the COS and CDS may require revision.

## Collaborators

- Project lead: West CT (Conceptualization, Data curation, Formal analysis, Funding acquisition, Investigation, Methodology, Project administration, Visualization, Writing—original draft).
- Supervisors: West MA, Mirnezami AH (Conceptualization, Methodology, Supervision, Writing—review & editing).
- Steering committee: Drami I, Glyn T, Sutton PA, Tiernan J, Behrenbruch C, Guerra G, Waters PS, Woodward N, Applin S, Charles SJ, Rose SA (Conceptualization, Methodology, Investigation, Resources, Writing—review & editing).
- Translation: Denys A, Pape E, van Ramshorst GH (Data curation, Investigation, Resources, Writing—review & editing).
- Patient representatives: Baker D, Bignall E, Blair I, Davis P, Edwards T, Jackson K, Leendertse PG, Love-Mott E, MacKenzie L, Martens F, Meredith D, Nettleton SE, Trotman MP, van Hecke JJM, Weemaes AMJ (Investigation).
- Delphi contributors: Abecasis N, Angenete E, Aziz O, Bacalbasa N, Barton D, Baseckas G, Beggs A, Brown K, Buchwald P, Burling D, Burns E, Caycedo-Marulanda A, Chang GJ, Coyne PE, Croner RS, Daniels IR, Denost QD, Drozdov E, Eglinton T, Espín-Basany E, Evans MD, Flatmark K, Folkesson J, Frizelle FA, Gallego MA, Gil-Moreno A, Goffredo P, Griffiths B, Gwenaël F, Harris DA, Iversen LH, Kandaswamy GV, Kazi M, Kelly ME, Kokelaar R, Kusters M, Langheinrich MC, Larach T, Lydrup ML, Lyons A, Mann C, McDermott FD, Monson JRT, Neeff H, Negoi I, Ng JL, Nicolaou M, Palmer G, Parnaby C, Pellino G, Peterson AC, Quyn A, Rogers A, Rothbarth J, Abu Saadeh F, Saklani A, Sammour T, Sayyed R, Smart NJ, Smith T, Sorrentino L, Steele SR, Stitzenberg K, Taylor C, Teras J, Thanapal MR, Thorgersen E, Vasquez-Jimenez W, Waller J, Weber K, Wolthuis A, Winter DC (Investigation).
- ACPGBI Committee: Branagan G, Vimalachandran D (Validation).
- PelvEx: Aalbers AGJ, Abdul Aziz N, Abraham-Nordling M, Akiyoshi T, Alahmadi R, Alberda W, Albert M, Andric M, Angeles M, Antoniou A, Armitage J, Auer R, Austin KK, Aytac E, Baker RP, Bali M, Baransi S, Bebington B, Bedford M, Bednarski BK, Beets GL, Berg PL, Bergzoll C, Biondo S, Boyle K, Bordeianou L, Brecelj E, Bremers AB, Brunner M, Bui A, Burgess A, Burger JWA, Campain N, Carvalhal S, Castro L, Ceelen W, Chan KKL, Chew MH, Chok AK, Chong P, Christensen HK, Clouston H, Collins D, Colquhoun AJ, Constantinides J, Corr A, Coscia M, Cosimelli M, Cotsoglou C, Damjanovic L, Davies M, Davies RJ, Delaney CP, de Wilt JHW, Deutsch C, Dietz D, Domingo S, Dozois EJ, Duff M, Egger E, Enrique-Navascues JM, Espín-Basany E, Eyjólfsdóttir B, Fahy M, Fearnhead NS, Fichtner-Feigl S, Fleming F, Flor B, Foskett K, Funder J, García-Granero E, García-Sabrido JL, Gargiulo M, Gava VG, Gentilini L, George ML, George V, Georgiou P, Ghosh A, Ghouti L, Giner F, Ginther N, Glover T, Golda T, Gomez CM, Harris C, Hagemans JAW, Hanchanale V, Harji DP, Helbren C, Helewa RM, Hellawell G, Heriot AG, Hochman D, Hohenberger W, Holm T, Holmström A, Hompes R, Hornung B, Hurton S, Hyun E, Ito M, Jenkins JT, Jourand K, Kaffenberger S, Kapur S, Kanemitsu Y, Kaufman M, Kelley SR, Keller DS, Kersting S, Ketelaers SHJ, Khan MS, Khaw J, Kim H, Kim HJ, Kiran R, Koh CE, Kok NFM, Kontovounisios C, Kose F, Koutra M, Kraft M, Kristensen HØ, Kumar S, Lago V, Lakkis Z, Lampe B, Larsen SG, Larson DW, Law WL, Laurberg S, Lee PJ, Limbert M, Loria A, Lynch AC, Mackintosh M, Mantyh C, Mathis KL, Margues CFS, Martinez A, Martling A, Meijerink WJHJ, Merchea A, Merkel S, Mehta AM, McArthur DR, McCormick JJ, McGrath JS, McPhee A, Maciel J, Malde S, Manfredelli S, Mikalauskas S, Modest D, Morton JR, Mullaney TG, Navarro AS, Neto JWM, Nguyen B, Nielsen MB, Nieuwenhuijzen GAP, Nilsson PJ, Nordkamp S, O’Dwyer ST, Paarnio K, Pappou E, Park J, Patsouras D, Peacock O, Pfeffer F, Piqeur F, Pinson J, Poggioli G, Proud D, Quinn M, Oliver A, Radwan RW, Rajendran N, Rao C, Rasheed S, Rasmussen PC, Rausa E, Regenbogen SE, Reims HM, Renehan A, Rintala J, Rocha R, Rochester M, Rohila J, Rottoli M, Roxburgh C, Rutten HJT, Safar B, Sagar PM, Sahai A, Schizas AMP, Schwarzkopf E, Scripcariu D, Scripcariu V, Seifert G, Selvasekar C, Shaban M, Shaikh I, Shida D, Simpson A, Skeie-Jensen T, Smart P, Smith JJ, Solbakken AM, Solomon MJ, Sørensen MM, Spasojevic M, Steffens D, Stocchi L, Stylianides NA, Swartling T, Sumrien H, Swartking T, Takala H, Tan EJ, Taylor D, Tejedor P, Tekin A, Tekkis PP, Thaysen HV, Thurairaja R, Toh EL, Tsarkov P, Tolenaar J, Tsukada Y, Tsukamoto S, Tuech JJ, Turner G, Turner WH, Tuynman JB, Valente M, van Rees J, van Zoggel D, Vásquez-Jiménez W, Verhoef C, Vierimaa M, Vizzielli G, Voogt ELK, Uehara K, Wakeman C, Warrier S, Wasmuth HH, Weiser MR, Westney OL, Wheeler JMD, Wild J, Wilson M, Yano H, Yip B, Yip J, Yoo RN, Zappa MA (Conceptualization).

## Supplementary Material

znae042_Supplementary_Data

## Data Availability

The data sets generated during and analysed during the current study are available from the corresponding author on reasonable request.

## Pre-registration

This study was pre-registered with an analysis plan at clinicaltrials.gov/NCT05683795

## Originality

This article is original work has not been published before, and is not under consideration for publication elsewhere. Republication of the data will not constitute redundant publication, will not breach copyright, and will reference the original publication.

## Previous communications

- PelvEx Amsterdam 2022 (PelvEx Initiative—The Empty Pelvis).
- PelvEx Bordeaux 2023 (Empty Pelvis after Pelvic Exenteration—Final Consensus meeting of the Delphi PelvEx Study).
- ACPGBI Manchester 2023 (‘Eureka’—objective assessment of the empty pelvis syndrome to measure volumetric changes in pelvic dead space following pelvic exenteration).
- ESCP Vilnius 2023 (A feasibility study of preoperative 3D volumetric pelvic measurement using automated DEEP-learning CT segmentation to better define the empty pelvis after pelvic exenteration surgery).

## Appendix

### Preparation, voting and discussion for measuring the core outcome set and core descriptor set

For each shortlisted outcome or descriptor, preparatory work is described in line with COSMIN guidance^35^, votes from questions raised at the face-to-face consensus meeting are given and a summary of any discussions are stated. To identify relevant systematic reviews a MEDLINE search on 24/05/2023 using the term ‘pelvic exenteration’ (PE) was performed, returning 28 results.

#### Empty pelvis syndrome core outcome set

##### Postoperative bowel obstruction

Definitions of bowel obstruction in the identified systematic reviews were inconsistent^57–59^. In relation to EPS, bowel obstruction has been defined as only within 90 days of surgery, and also over 90 days specifying a transition point in the pelvis^1,3,26^. CT can diagnose bowel obstruction with a sensitivity of 94% and specificity of 96%, and can identify transition points^60^.

- Should diagnosis of small bowel obstruction due to EPS be on CT with a transition point in the pelvis?
  *65/70 (92.9%) yes, 2/70 (2.86%) no, 3/70 (4.29%) unsure*
- Should the core outcome set only capture small bowel obstruction within 90 days of surgery, or over 90 days?
  *Over 90 days 67/77 (87.0%), under 90 days 5/77 (6.49%), 5/77 (6.49%) unsure*

##### Enteroperineal fistula

Systematic reviews used inconsistent terms including chronic fistulas, enterocutaneous fistula, enterovesical fistula, enteric fistula, perineal fistula and fistula^1,58,59,61,62^.

- Should only an enteric fistula arising from small bowel within the pelvis be reported?
  *72/79 (91.1%) yes, 7/79 (8.86%) no*
- Should fistula from small bowel within the pelvis, into other viscera, or remnants of other viscera be reported? That is, enteric fistulation through a vaginal suture line.
  *83/86 (96.5%) yes, 3/86 (3.49%) no*

Discussion pointed out that the statement should be fistulation from any bowel within the pelvis including the caecum, and not just small bowel. There was some uncertainty whether an enteroperineal fistula occurs due to EPS or whether this is due to the effect of gravity.

#### Quality of life

Six systematic reviews focusing on quality of life following PE have been performed, describing 40 different PROMs, and time points for collection of PROMs is between baseline and 8 years post-surgery^63–68^. This includes a systematic review using the COSMIN risk of bias checklist for PROMs in patients with locally recurrent rectal cancer; in this paper, appropriate content validity was not identified in any individual PROM^63^. A validated PROM for locally recurrent rectal cancer is now in use; however, it is not validated for patients undergoing PE for other indications^69^. PelvEx are already supporting design of a specific PE PROM through the European Organisation for Research and Treatment of Cancer^20^.

- Should further validation work be completed to develop a PelvEx PROM for patients undergoing PE?
  *80/83 (96.4%) yes, 1/83 (1.20%) no, 2/83 (2.41%) unsure*

##### Rate of return to theatre and use of interventional radiology to manage complications of EPS

Systematic reviews describe reoperation rates and the need for interventional radiology to manage complications after PE; reasons for re-intervention are usually given^1,57,62,70,71^. The Clavien–Dindo classification is used to report these interventions, this method being well established and encompassing radiological intervention^72^. There is also persistent lack of consensus on how to manage complications of EPS; however, this was felt to be beyond the scope of this study in the planning phase; reporting detail on procedures performed to manage issues would assist in building a body of evidence to address this^3^.

- Should Clavien–Dindo classification be applied to any EPS complication?
  *69/73 (94.5%) yes, 1/73 (1.37%) no, 3/73 (4.11%) unsure*
- Should the reason for re-intervention be stated?
  *62/66 (93.9%) yes, 1/66 (1.52%) no, 3/66 (4.55%) unsure*
- Should the procedure performed for the complication be stated?
  *66/70 (94.3%) yes, 1/70 (1.43%) no, 3/70 (4.29%) unsure*

##### Chronic perineal sinus

Chronic perineal sinuses were not defined in the systematic reviews and were described as sinus formation, chronic sinuses and chronically draining perineal wounds^1,57,61^. In the context of abdominoperineal resection, a persistent perineal sinus has been defined as a perineal wound remaining unhealed for more than 6 months; this definition has persisted for over 50 years^73,74^. Following PE, fluid may also discharge from the empty space through the remnant pelvic viscera, such as a vaginal suture line.

- Should a persistent perineal sinus in the context of EPS be defined as fluid discharging from a perineal wound remaining unhealed for more than 6 months?
  *65/67 (97.0%) yes, 1/67 (1.49%) no, 1/67 (1.49%) unsure*
- Should perineal discharge due to a connection between an empty pelvic cavity and remnant pelvic viscera that has been present for over 6 months also be reported?
  *71/77 (92.2%) yes, 5/77 (6.49%) no, 1/77 (1.30%) unsure*

##### Flap-related morbidity

Systematic reviews report flap morbidity as major complications (interventional required), minor complications (conservative treatment only) and include donor site morbidity^70^, otherwise specific recipient site complications are given^71^. PelvEx 8 is already underway, investigating perineal flap reconstruction following surgery for advanced pelvic malignancy^75^.

- Should the same outcomes as PelvEx 8 be used?
  *64/75 (85.3%) yes, 4/75 (5.33%) no, 7/75 (9.33%) unsure*
- Should complications on donor site morbidity also be reported?
  *42/71 (59.2%) yes, 24/71 (33.8%) no, 5/71 (7.04%) unsure*
- Should prosthesis failure or removal be reported?
  *62/69 (89.9%) yes, 5/69 (7.25%) no, 2/69 (2.90%) unsure*

Flap-related morbidity was discussed at more length, requiring two votes before being included into the final core outcome set. Polarized views were that flap morbidity was not directly related to EPS and was more likely to be an issue with blood supply or technique, and that some centres tended to use mesh or other implants to mitigate EPS; therefore, this would not be applicable to them.

Counterarguments were that flaps are performed to address EPS, and therefore assessing morbidity of this would be important, or why do them? In addition, it was felt that EPS complications, such as enteroperineal fistula or infected pelvic collections, were likely to affect flap outcome. Several flaps may need to be raised in a composite manner for more complex PE with an ‘emptier’ pelvis; this would likely cause additional flap morbidity. It was then established that this outcome should encompass morbidity from any reconstruction used to mitigate the EPS, and therefore should include complications from the use of implants to mitigate EPS, thereby giving further insight into prevention of EPS.

##### Infected postoperative pelvic fluid collections or pelvic abscess

Systematic reviews use the terms perineal abscess, deep perineal abscess, pelvic abscess, perineal wound abscess, pelvic collection, abscess and pelvic sepsis^57,58,61,62,71^. In the context of PE, the boundary between pelvis and perineum may be resected and reconstructed; therefore, post-surgery a pelvic abscess may be difficult to distinguish from a perineal abscess. Infected pelvic collections directly attributed to EPS are reported as being within 30 days of surgery, but also beyond this^1,3,26^.

CT is well established in diagnosis of postoperative intra-abdominal sepsis; however, an infected pelvic fluid collection is not well defined^76^. Four scoring systems are published to distinguish infected from non-infected abdominal fluid collections post-surgery^77–80^. These were identified using MEDLINE and EMBASE searches on 25 May 2023 using the term ‘infected postoperative fluid CT’, which identified 633 results. All of these scoring systems were feasible, and the ClinROM COSMIN risk of bias of tool was used to assess these instruments^45^. The scoring system by Gnannt *et al.* (2015)^79^ was rated very good and was also externally validated; the other three were rated overall doubtful or inadequate and had not been externally validated. Practically radiologists provide reports on CTs, diagnosing infected pelvic collections using their own judgement and on discussion with clinical teams.

The longlisted statements on urological outcomes, including urinary leakage and anastomotic leakage, did not make it through the shortlisting stage. Leaks may also manifest as pelvic collections despite not being a defined part of EPS.

- Should any abscess within the pelvis or neo-perineum be reported?
  *66/68 (97.1%) yes, 2/68 (2.94%) no*
- Should CT be used to report this complication?
  *58/60 (96.7%) yes, 1/60 (1.67%) no, 1/60 (1.67%) unsure*
- Should diagnosis be on the basis of a radiologist's report or other scoring system?
  *Radiologist's report 61/68 (89.7%), scoring system 6/68 (8.82%), unsure 1/68 (1.47%)*
- Should infected pelvic fluid collections be recorded only up to 30 days or beyond this?
  *Over 30 days by 51/70 (72.9%), under 30 days by 19/70 (27.1%)*
- Should patients with collections secondary to intestinal or urinary anastomotic leakage be excluded from studies on EPS?
  *24/74 (32.4%) yes, 41/74 (55.4%) no, 9/74 (12.2%) unsure*

There was uncertainty whether a leak from an anastomosis, such as ileal conduit, would occur anyway or whether it would be more likely if pulled down into an empty pelvic space. There was criticism, however, on excluding anastomotic leakage completely, as identifying causality by excluding factors that cannot be perfectly defined will confound interpretation of results. It was therefore decided to include collections secondary to anastomotic leakage; however, if leaks are known to have occurred then they should be specified.

#### Empty pelvis syndrome core descriptor set

##### Radiotherapy-induced damage

Neoadjuvant external beam radiotherapy is described variably as long- or short-course radiotherapy in rectal cancers^57,61,62^, binary with patients either ‘receiving’ or ‘not receiving’ radiotherapy^58,70,71,81^, using centigray (cGy)^62,82,83^, or Gray (Gy)^57,62^. In the PE population Gy is used to describe radiotherapy doses in rectal, anal and cervical cancers^82–84^. Intraoperative radiotherapy treatments are also reported separately to external beam radiotherapy, again either with a binary yes/no, or using Gy^58,62,84^. The absorption of Gy to the emptied pelvis using these different modalities will be different.

- Should radiotherapy be reported using Gy, or ‘yes received’/‘no did not receive’?
  *Measure with Gy voted by 61/69 (88.4%), measure with binary yes or no 6/69 (8.70%), 2/69 (2.90%) were unsure*
- If Gy, should the total cumulative dosage to the pelvis that a patient has had be reported?
  *57/63 (90.5%) yes, 1/63 (1.59%) no, 5/63 (7.94%) unsure*
- Should the use of different radiotherapy modalities be reported separately; that is, external beam radiotherapy *versus* intraoperative electron radiotherapy *versus* brachytherapy?
  *53/64 (82.8%) yes, 7/64 (10.9%) no, 4/64 (6.25%) unsure*

##### The greater the magnitude and radicality of surgery the worse the complications from the empty pelvis syndrome will be

Classification of the radicality of PE is inconsistent, with terminology used including total PE, anterior PE, posterior PE, soft tissue exenteration, partial PE, supralevator PE, infralevator PE, beyond total mesorectal excision, extended resection, wide tumour resection, colectomy and *en-bloc* resection of at least one other organ, extended proctectomy and modified PE^1,57–59,61,62,64,65,67,68,84–87^. Further detail may be given on additional resections, but these are imprecise descriptions, including: *en-bloc* bony resection and excision of major sacral nerve^68^; sacrectomy^1^; high or low sacrectomy^81^; central compartment, anterior compartment, sphincter preservation, lateral lymph node dissection^57^; neurovascular structures, multiple pelvic compartments^59^; and extended bony resection^87^. Definitions for exenterative terms are inconsistent:

- PE: excision of tumour mass (including rectum or neo-rectum) and at least one adjacent organ^61^; *en-bloc* resection of rectum, genitourinary viscera, reproductive internal organs, regional lymph nodes and peritoneum^1^; a cluster of several surgical procedures that are challenging to standardize^64^; colectomy and *en-bloc* resection of one or more organs^59^; or resection of primary or recurrent tumour with three or more structures across two or more pelvic compartments^66^.
- Total PE: all internal pelvic organs^65^; complete visceral exenteration with two stomas^1^; rectum, bladder, prostate/uterus/vagina, lateral pelvic lymph nodes^57^; or all pelvic organs with resections often outside the pelvic box into bones, muscles and neurovascular components of sidewall^88^.
- Posterior PE: uterus and rectum^58,65^; or partial/total vaginectomy with rectosigmoid resection^88^.
- Anterior PE: bladder and uterus ± adnexa^65^; bladder, uterus and upper rectosigmoid with sparing of lower rectum and perineum^57,58^; or partial or total vaginectomy with bladder and urethra resected^88^.

One systematic review does classify exenterations in the context of PE for gynaecological cancers; however, detail on how the system was formulated is not given and it is not within common use^88^. To identify potential measurement instruments a search on MEDLINE and EMBASE was performed on 26 May 2023 with the term ‘pelvic exenteration classification’—206 results were found, and two papers described methodological design of classification systems for PE^47,50^. Laporte *et al.* (2020)^47^ was rated overall inadequate, and Burns *et al*. (2023)^50^ was rated overall adequate with the COSMIN risk of bias tool^45^.

- Should the PE lexicon be used to standardize reporting of magnitude of resection?
  *64/67 (95.5%) yes, 1/67 (1.49%) no, 2/67 (2.99%) unsure*

##### Methods of reconstruction to fill the pelvis influences development of the empty pelvis syndrome

Systematic reviews described primary closure, flap, mesh, myocutaneous flap, omental flap, breast prosthesis, silicone expanders, obstetric balloons, biological mesh, gluteal flap, rectus flaps, colorectal anastomosis, colonic flap, upper thigh flap or perineal flaps for reconstruction^1,57,58,61,62,70,71^.

- Should detail on the techniques or prostheses used to fill the empty pelvis be reported?
  *66/66 (100%) yes*

##### Changes in pelvic dead space following exenteration

Shortlisted statements ‘A lack of pelvic filling leads to different complications to those relating to the perineal wound’ and ‘Small bowel falling into the pelvic cavity contributes to EPS’ could be quantified by measuring volumetric changes in the pelvic dead space before and after PE. Two studies were identified that calibrated the size of the empty pelvic cavity following PE to measure the size of appropriate breast implants for pelvic filling and reduction of dead space; however, these were not feasible and were inadequate using the COSMIN risk of bias tool^13,45,89^.

- Should further validation work be performed to see if volumetric changes in pelvic dead space reliably correlate to complications of EPS?
  *39/54 (72.2%) yes, 9/54 (16.7%) no, 6/54 (11.1%) unsure*
