# Corrigendum: The empty pelvis syndrome: a core data set from the PelvEx collaborative

**DOI:** 10.1093/bjs/znae095

**Published:** 2024-03-29

This is a corrigendum to: PelvEx Collaborative, The empty pelvis syndrome: a core data set from the PelvEx collaborative, *British Journal of Surgery*, Volume 111, Issue 3, March 2024, znae042, https://doi.org/10.1093/bjs/znae042

In the originally published version of the manuscript there was a typographical error in the name of one of the collaborative authors. This should read: “ACPGBI Committee: Branagan G,[…]”.

The emendation has been made to the article.

