# Supplementary material for: The empty pelvis syndrome: a core data set from the PelvEx collaborative
Source: Br J Surg. 2024 Mar 8;111(3):znae042. doi: 10.1093/bjs/znae042 (PMC10921833; doi:10.1093/bjs/znae042)
Supplement: znae042_Supplementary_Data [file znae042_supplementary_data.zip › Figure_S1.docx]

A


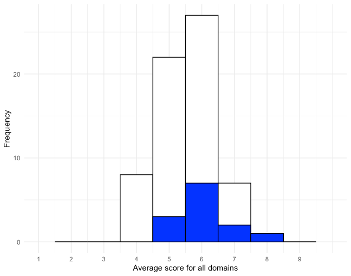


B


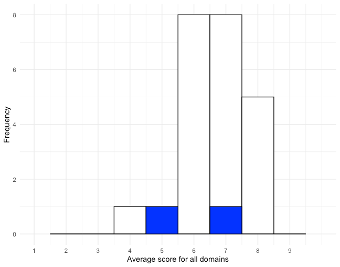


Figure S1 – Attrition analysis for healthcare professionals in A and patient representatives in B, average scores for all domains in round one are shown for individuals completing rounds one and two in white, with those only completing round one in blue.
