# Supplementary material for: The empty pelvis syndrome: a core data set from the PelvEx collaborative
Source: Br J Surg. 2024 Mar 8;111(3):znae042. doi: 10.1093/bjs/znae042 (PMC10921833; doi:10.1093/bjs/znae042)
Supplement: znae042_Supplementary_Data [file znae042_supplementary_data.zip › Table_S1.docx]

| **Authors** | **Pathophysiology** | **Outcomes** |
| --- | --- | --- |
| Barber, et al. 1993^2^ | Flu-like illness, particularly in those undergoing heavy irradiation.  Denuded pelvis, and an empty pelvic space. | Malaise, elevated temperature, increased discharge from the perineal sinus that may continue for many years. |
| Robertson, et al. 1994^5^ | Nil specified | Nil specified |
| Finan, et al. 1996^6^ | Flu-like illness. | Fever, lethargy, serous drainage from a chronic pelvic/perineal sinus. |
| Palfalvi, 1998^8^ | Following evisceration of pelvic organs small bowel adheres to the denuded and irradiated pelvic floor.  Previous irradiated small bowel loops are implicated. | Small bowel obstruction, fistula formation, perineal herniation. |
| Ghosh, et al. 2004^7^ | Empty pelvic cavity following exenteration with or without chronic infection. | Abscess, haematoma, lymphocele leading to persistent drainage and sinus formation. |
| Bacalbasa, et al. 2015^9^ | Large defect at the level of the pelvic diaphragm with visceral herniation of small bowel in a pre-irradiated, hypoxic or denuded pelvic floor. | Enteroperineal fistula. |
| Ferron, et al. 2015^10^ | Reduced vascularity and increased dead space. | Pelvic abscess and fistulae. |
| de-la-Noval, et al. 2017^30^ | Empty cavity following pelvic exenteration. | Small bowel obstruction, fistula formation, visceral or perineal herniation, recurrent pelvic abscesses, haematoma, or lymphocele; leading to chronic discharge and infections. |
| Continovis, et al. 2018^11^ | Dead space in the pelvis, and severe adhesions to denuded pelvic sidewall. | Abscess formation, bowel obstructions, continuous discharge, bowel perforation, and fistulas. |
| Lee, et al. 2019^12^ | Void created after pelvic exenteration leads to pelvic fluid accumulation and small bowel translocation. | Pelvic abscess, perineal fluid discharge, perineal wound dehiscence, and prolonged ileus. |
| Carboni, et al. 2019^13^ | Residual dead space following pelvic organ excision causing a large poorly vascularized space, with radiation-induced bowel injury implicated. | Abscess formation, bowel obstruction, perforation, and enterocutaneous fistulas. |
| Lau, et al. 2019^14^ | Empty space that remains following complete soft tissue exenteration promotes collection of fluid and adherence of small bowel to the denuded pelvis.  Particularly problematic with en bloc major bony resection where cut edges of exposed bone are present | Abscess formation, discharge from the perineal wound, infection and dehiscence. |
| Tuech, et al. 2020^15^ | Empty cavity following pelvic exenteration resulting in fluid accumulation within the pelvis.  Irradiated small bowel loops and enterostomy may become adherent to exposed pelvic surfaces. | Pelvic abscess, perineal fluid discharge, perineal wound dehiscence, prolonged ileus, bowel obstruction and enteroperineal fistula. |
| Bankar, et al. 2020^16^ | Large empty space left in the pelvis | Post-operative collections, abscess formation, prolonged ileus, intestinal obstruction, and small bowel fistulas. |
| Manzour, et al. 2021^17^ | Nil specified | Post-operative collections, abscess formation, prolonged ileus, intestinal obstruction, and small bowel fistulas. |
| Ng, et al. 2021^18^ | Large tissue defects causing a void left in the pelvis, with wide pelvic floor excision.  Prior irradiation of soft tissue impeding healing, potential devascularisation of perineal skin flaps if internal iliac vessels are ligated.  Exposure of raw bone surfaces.  Fluid accumulation and translocation of bowel into the dependent empty pelvic cavity. | Perineal herniae, complex fistula, pelvic abscess, perineal fluid discharge, perineal wound dehiscence, and prolonged ileus. |
| Martinez-Gomez, et al. 2021^25^ | Empty space left in the pelvis. | Fistula, pelvic collection, chronic infection, osteomyelitis, and organ prolapse. |
| Wang, et al. 2021^19^ | Pelvic dead space after pelvic exenteration  Absence of mechanical support after removal of the pelvic floor and pelvic organs allowing any intra-abdominal contents to descend, together with bowel adhesions deep in the denuded pelvis.  Post-operative radiotherapy then increases risk of chronic radiation enteritis.  Small bowel translocation can cause perineal herniation.  Void left after radical resection leads to pelvic fluid accumulation.  Downward pressure of descending intra-abdominal contents. | Abscess formation, bowel obstruction, haematoma, fistulisation, perineal herniation, persistent discharge, wound infection, and wound dehiscence. |
| PelvEx Collaborative, 2022^20^ | Dead space allows for accumulation of fluid and small bowel migration into the pelvis. | Bowel obstruction |
| Omarov, et al. 2022^21^ | Due to pelvic dead space. | Haematoma, abscess, permanent pus discharge, chronic infections, bowel obstruction, perforation, and enterocutaneous fistulas. |
| Persson, et al. 2022^22^ | Resection of pelvic organs leaving a ‘dead space’ in the pelvis. | Pelvic collections, perineal wound complications, small bowel adhesions and small bowel fistulae. |
| Wang, et al. 2022^23^ | Pelvis fills with fluid which can become infected.  Worse in smokers and diabetic patients. | Collections, hampered healing, perineal wound breakdown, chronic abscess, and fistula formation. |
| Shine, et al. 2022^24^ | Reasons are multifactorial including irradiated and scarred soft tissue, partial devascularisation of skin flaps due to high ligation of internal iliacs, large tissue defects, exposed bone and irradiated small bowel. | Perineal wound breakdown, perineal hernias and fistulas |
| Kazi, et al. 2022^26^ | Pelvic void created after evisceration of the pelvis  Sequelae of EPS not limited to within 90 days, with complications occurring months and years later. | Early and late morbidity, pelvic collection, small bowel obstruction, enteric fistula, perineal hernia, perineal wound dehiscence, and perineal wound infections. |
| Sutton, et al. 2022^3^ | Probably relates to the void left after pelvic evisceration, relocation of bowel into the pelvis, significant impairment of the pelvic floor due to wide excision, and impaired tissue healing due to multifactorial chronic ischaemia.  Proximal ligation of the internal iliacs, scar tissue from previous surgery, radiation induced fibrosis, and exposure of denuded bony surfaces may all contribute to chronic ischaemia.  Fluid accumulation within the empty devascularised cavity.  Translocation of small bowel may adhere to exposed bony surfaces.  Radiation enteritis.  Development of long-term EPS complications are more likely with a greater anatomical extent of resection. | Perineal wound breakdown, perineal herniation, complex fistulas, abscess formation, perineal fluid discharge, wound dehiscence, chronic discharge, obstruction, and prolonged ileus. |
| Khaw, et al. 2022^27^ | Creation of an empty space, which has the potential to fill with fluid or bowel.  Complications can be acute to chronic and longstanding.  The simultaneously created dead space leads to bowel translocation into the pelvis and fluid collections to an anatomically weakened pelvic floor. | Pelvic collections, bowel obstruction, fistula, perineal wound dehiscence, perineal wound leakage, delayed healing, chronic discharge, infection, and herniation. |
| Manzour, et al. 2022^17^ | Empty space created after surgery leads to fluid accumulation and small bowel adherence to the pelvic floor. | Fistula formation, pelvic abscess, or chronic infection. |
| Aminimoghaddam, et al. 2022^29^ | Occurs due to vacant space in the pelvic cavity after total pelvic exenteration.  Collection of pelvic fluid and descending of the intestinal loops in the pelvic cavity. | Sepsis, wound complications, bowel obstruction, fistulas, and fluid collections. |
| Johnson, et al. 2022^1^ | A large defect or void generated following pelvic exenteration.  Due to the sequelae of fluid accumulation and migration of small bowel into the empty pelvis. | Pelvic abscess, collection, prolonged ileus, mechanical bowel obstruction, fistula, and sinus formation. |

Table S1 – Publications on the empty pelvis syndrome, empty pelvic syndrome and pelvic burn syndrome. References were found using the search terms “empty pelvi*”, and “pelvic burn syndrome’ on MEDLINE and EMBASE on 09/03/2023, followed by searching citations. Shown are the pathophysiological factors and outcomes that each paper reports for EPS.
