# Supplementary material for: The empty pelvis syndrome: a core data set from the PelvEx collaborative
Source: Br J Surg. 2024 Mar 8;111(3):znae042. doi: 10.1093/bjs/znae042 (PMC10921833; doi:10.1093/bjs/znae042)
Supplement: znae042_Supplementary_Data [file znae042_supplementary_data.zip › Table_S2.docx]

| **Empty Pelvis Syndrome**  **Core Outcome Set Longlisted Statements** | **Patient Representative Voting (%)** | | **Healthcare Professional Voting (%)** | | **Consensus** |
| --- | --- | --- | --- | --- | --- |
|  | **Votes 1 - 3** | **Votes 7 - 9** | **Votes 1 - 3** | **Votes 7 - 9** |  |
| Perineal wound dehiscence rate | 0 | 50 | 10.3 | 64.1 | In |
| Post-operative bowel obstruction | 4.17 | 58.3 | 3.85 | 67.9 | In |
| Enterocutaneous fistula formation | 8.33 | 62.5 | 1.30 | 75.3 | In |
| Perineal hernia rate | 8.33 | 41.7 | 7.69 | 59.0 | In |
| Length of stay for index admission | 8.33 | 54.2 | 19.2 | 46.2 | In |
| Rate of return to theatre for EPS | 8.33 | 58.3 | 1.28 | 73.1 | In |
| Quality of life using patient reported outcome measures | 0 | 83.3 | 1.28 | 80.8 | In |
| Perineal wound infection rate | 8.33 | 45.8 | 12.8 | 53.8 | In |
| Intestinal anastomotic leak rate | 8.33 | 54.2 | 16.7 | 52.6 | In |
| Specific urological outcomes | 4.17 | 70.8 | 10.3 | 51.3 | In |
| Post-operative mortality rate | 20.8 | 58.3 | 7.69 | 67.9 | In |
| Volumetric assessments to objectively measure how filled or unfilled the pelvis is after exenteration | 4.17 | 58.3 | 17.9 | 44.9 | In |
| Chronic perineal sinus rate | 4.17 | 50 | 7.69 | 64.1 | In |
| Infected post-operative pelvic fluid collection | 8.33 | 58.3 | 5.13 | 64.1 | In |
| Health economic impact | 33.3 | 25 | 15.9 | 50 | Out |
| Measurement of prolonged ileus | 16.7 | 33.3 | 16.7 | 32.1 | Out |
| Re-admission rate for EPS | 12.5 | 33.3 | 1.28 | 62.8 | In |
| Utilisation of a common exenterative lexicon to standardise reporting of the radicality of surgery | 8.33 | 58.3 | 3.85 | 70.5 | Merged (following thematic analysis) |
| Information sheets on complications after pelvic exenteration including on EPS | 4.17 | 70.8 | 14.1 | 37.2 | Out (following thematic analysis) |
| **Empty Pelvis Syndrome Pathophysiology Domain Longlisted Statements** |  |  | **Healthcare Professional Voting (%)** | | **Consensus** |
|  |  |  | **Votes 1 – 3** | **Votes 7 – 9** |  |
| A lack of pelvic filling after exenteration leads to distinct complications from problems relating to the perineal wound |  |  | 5.19 | 68.8 | In |
| Accumulation of serous and lymphatic fluid into the empty pelvis from dissected tissues contributes to EPS |  |  | 15.9 | 53.2 | Out |
| Complications of EPS will be worse after minimally invasive techniques due to a reduced formation of adhesions, when compared to open surgery |  |  | 36.4 | 13.0 | Out |
| Dissection causing denuded exposed bone contributes to EPS |  |  | 15.6 | 41.6 | Out |
| Dissection through fibrotic scar tissue in cases of recurrent disease leading to worsening relative regional ischaemia contributes to EPS |  |  | 16.9 | 24.7 | Out |
| Exenterative surgery causing an anatomically weakened pelvic floor contributes to EPS |  |  | 9.09 | 63.6 | In |
| Most of the morbidity from EPS occurs in a chronic manner after 3 months from surgery |  |  | 16.9 | 44.2 | Out |
| Most of the morbidity from EPS occurs in the immediate post-operative period within 3 months of surgery |  |  | 19.5 | 44.2 | Out |
| Patients with significant adhesions at the time of pelvic exenteration, for example in recurrent cases, will have less morbidity from EPS |  |  | 33.8 | 22.1 | Out |
| Patients that undergo non-exenterative total mesorectal excision, hysterectomy, or cystectomy will also suffer from EPS, but to a lesser degree |  |  | 15.6 | 54.5 | Out |
| Radiation induced fibrosis leading to relative ischaemia contributes to EPS |  |  | 14.3 | 55.8 | Merged following thematic analysis |
| Radiotherapy causing enteritis leads to a higher chance of empty pelvis complications |  |  | 10.4 | 50.6 | Merged following thematic analysis |
| Sacrifice of internal iliac system vessels leading to regional arterial or venous ischaemia contributes to EPS |  |  | 28.6 | 39.0 | Out |
| Small bowel translocation into the empty pelvis contributes to EPS |  |  | 6.49 | 72.7 | In |
| The greater the magnitude and radicality of surgery the worse the complications from EPS will be |  |  | 3.90 | 72.7 | In |
| The patient’s gender is an anatomical risk factor for developing EPS independent of radicality of surgery |  |  | 20.8 | 40.3 | Out |
| Patient co-morbidities have an independent importance for developing EPS |  |  | 10.4 | 46.7 | Out |
| **Empty Pelvis Syndrome Mitigation Domain Longlisted Statements** |  |  | **Healthcare Professional Voting** | | **Consensus** |
|  |  |  | **Votes 1 – 3**  **(%)** | **Votes 7 – 9 (%)** |  |
| A permanent prosthetic device to fill the pelvis |  |  | 14.9 | 42.0 | Out |
| A post-partum haemorrhage obstetric balloon placed to fill the pelvis at the end of surgery, with subsequent deflation and removal |  |  | 45.9 | 9.46 | Out |
| A saline-filled breast prosthesis placed to fill the pelvis at the end of surgery |  |  | 50 | 13.5 | Out |
| A silicone breast prosthesis placed to fill the pelvis at the end of surgery |  |  | 54.1 | 10.8 | Out |
| Dissection and mobilisation of remaining pelvic peritoneum to allow closure of the peritoneum and exclude the pelvis |  |  | 45.9 | 20.3 | Out |
| Early drainage of post-surgery collections that are not controlled with operatively placed drains within the pelvis |  |  | 16.2 | 48.6 | Out |
| Mobilisation of other structures to fill or exclude the empty pelvis from small bowel, i.e., caecum, bladder, uterus, or spare bowel mesentery |  |  | 13.5 | 60.8 | In |
| No deliberate manoeuvres used to fill the pelvis |  |  | 54.1 | 21.6 | Out |
| Performing primary closure of the skin and remaining levator muscle as the only reconstruction |  |  | 45.9 | 24.3 | Out |
| Placement of a mesh at the pelvic brim to act as a sling for the small bowel |  |  | 39.2 | 14.9 | Out |
| Placement of a mesh at the pelvic outlet to reinforce the remaining pelvic floor |  |  | 35.1 | 20.3 | Out |
| Plication of a loop of small bowel onto the pelvic brim in order to prevent other small bowel loops falling into the pelvis |  |  | 60.8 | 2.70 | Out |
| The continued prophylactic use of post-operative antibiotics to prevent infected fluid collections |  |  | 55.4 | 13.5 | Out |
| The use of 3D printed bioscaffolds to fill the pelvis |  |  | 31.1 | 14.9 | Out |
| The use of a bulky myocutaneous flap in order to achieve some pelvic filling |  |  | 5.41 | 78.4 | In |
| The use of a mesh made from a collagen tissue matrix |  |  | 27.0 | 21.6 | Out |
| The use of a mesh made from absorbable synthetic material |  |  | 40.5 | 12.2 | Out |
| The use of a mesh made from non-absorbable synthetic material |  |  | 58.1 | 12.2 | Out |
| The use of a muscle sparing flap to fill the pelvis |  |  | 13.5 | 41.9 | Out |
| The use of a muscle-only flap to fill the pelvis |  |  | 5.41 | 54.1 | In |
| The use of a non-bulky myocutaneous flap in order to achieve perineal coverage |  |  | 1.49 | 41.8 | Out |
| The use of a number of inflated Foley catheters placed into the empty pelvis and deflated over time |  |  | 59.5 | 5.41 | Out |
| The use of a pelvic drain for a fixed amount of time, for example 5 days |  |  | 45.9 | 16.2 | Out |
| The use of a pelvic drain until the output is negligible |  |  | 23.0 | 39.2 | Out |
| The use of a perineal drain for a fixed amount of time, for example 5 days |  |  | 48.6 | 16.2 | Out |
| The use of a perineal drain until output is negligible |  |  | 33.8 | 35.1 | Out |
| The use of a short course of prophylactic antibiotics to reduce risk of infected collections (less than 48 hours) |  |  | 45.9 | 20.3 | Out |
| The use of an omentoplasty for pelvic filling |  |  | 5.41 | 67.6 | In |
| The use of autologous fat transfer to make structures that are filling the pelvis bulkier, for example transfer of abdominal wall fat into an omentoplasty |  |  | 35.1 | 12.2 | Out |
| Tight control of post-operative nutrition with early or routine use of total parenteral nutrition |  |  | 24.3 | 39.2 | Out |
| Use of a silicone tissue expander placed into the empty pelvis and deflated over time |  |  | 41.9 | 13.5 | Out |
| Use of deliberate techniques to encourage the small bowel to form adhesions so it does not fall into the pelvis |  |  | 60.8 | 8.11 | Out |
| Use of multiple techniques to ensure the pelvis is adequately filled |  |  | 12.2 | 63.5 | In |
| Use of negative pressure dressings to reduce complications resulting from EPS |  |  | 36.5 | 25.7 | Out |

Table S2 – Longlisted statements, their corresponding votes from the first Delphi round, and whether they progressed, were dropped, or merged following thematic analysis; EPS – empty pelvis syndrome.
