# Supplementary material for: The empty pelvis syndrome: a core data set from the PelvEx collaborative
Source: Br J Surg. 2024 Mar 8;111(3):znae042. doi: 10.1093/bjs/znae042 (PMC10921833; doi:10.1093/bjs/znae042)
Supplement: znae042_Supplementary_Data [file znae042_supplementary_data.zip › Table_S3.docx]

| **Empty Pelvis Syndrome**  **Core Outcome Set Longlisted Statements** | **Patient Representative Voting (%)** | | **Healthcare Professional Voting (%)** | | **Consensus** |
| --- | --- | --- | --- | --- | --- |
|  | **Votes 1 - 3** | **Votes 7 - 9** | **Votes 1 - 3** | **Votes 7 - 9** |  |
| Perineal wound dehiscence and delayed perineal wound healing* | 4.35 | 60.9 | 2.28 | 64.6 | Out |
| Post-operative bowel obstruction | 4.35 | 69.6 | 1.05 | 87.7 | In |
| Enterocutaneous fistula formation | 4.35 | 65.2 | 1.04 | 89.2 | In |
| Perineal hernia rate | 8.70 | 60.9 | 2.34 | 58.5 | Out |
| Length of stay for index admission | 8.70 | 69.6 | 6.08 | 49.2 | Out |
| Rate of return to theatre and use of interventional radiology to manage complications of EPS* | 0 | 82.6 | 0 | 89.2 | In |
| Quality of life through validated patient reported outcome measures | 4.35 | 91.3 | 1.03 | 90.8 | In |
| Perineal wound infection rate | 8.70 | 56.5 | 4.59 | 63.1 | Out |
| Intestinal anastomotic leak rate | 8.70 | 47.8 | 8.35 | 53.8 | Out |
| Specific urological outcomes | 8.70 | 60.9 | 5.93 | 55.4 | Out |
| Post-operative mortality rate | 21.7 | 73.9 | 5.57 | 70.8 | In |
| Volumetric radiological objective assessments to measure differences in ratios of pelvic dead space before and after exenteration | 0 | 52.2 | 7.16 | 53.8 | Out |
| Chronic perineal sinus rate | 0 | 65.2 | 0 | 75.4 | In |
| Infected post-operative pelvic abscess or pelvic fluid collection* | 0 | 78.3 | 1.06 | 84.6 | In |
| Re-admission rate for EPS | 8.70 | 47.8 | 2.36 | 56.9 | Out |
| Development of osteomyelitis (NEW) | 4.35 | 60.9 | 5.89 | 56.9 | Out |
| Flap-related morbidity (NEW) | 4.35 | 65.2 | 1.11 | 72.3 | In |
| Measures of markers of metabolism and blood chemistry following surgery (NEW) | 0 | 39.1 | 16.3 | 24.6 | Out |
| **Empty Pelvis Syndrome Pathophysiology Domain Longlisted Statements** |  |  | **Healthcare Professional Voting (%)** | | **Consensus** |
|  |  |  | **Votes 1 – 3** | **Votes 7 – 9** |  |
| A lack of pelvic filling after exenteration leads to distinct complications from problems relating to the perineal wound |  |  | 0 | 87.7 | In |
| Accumulation of fluid into the empty pelvic cavity after exenteration contributes to the empty pelvis syndrome (NEW) |  |  | 6.00 | 52.3 | Out |
| Exenterative surgery causes an anatomically weakened pelvic floor, which contributes to EPS |  |  | 2.24 | 69.2 | Out |
| Radiation induced damage contributes to the development of the empty pelvis syndrome* |  |  | 1.06 | 83.1 | In |
| Small bowel falling into the empty pelvic cavity contributes to EPS* |  |  | 1.05 | 87.7 | In |
| EPS is multi-factorial and unpredictable (NEW) |  |  | 4.43 | 72.3 | In |
| The greater the magnitude and radicality of surgery the worse the complications from EPS will be |  |  | 0 | 87.7 | In |
| The main goals of pelvic reconstruction should be recorded (NEW) |  |  | 2.28 | 64.6 | Out |
| The methods of reconstruction used to ‘fill’ the empty pelvis following exenteration influences the development of EPS (NEW) |  |  | 0 | 90.8 | In |
| The presence of anastomoses and staple lines within the pelvis influences development of EPS (NEW) |  |  | 4.86 | 49.2 | Out |
| The type / anatomical point of origin of the tumour that patient has, is important in development of EPS (NEW) |  |  | 5.81 | 60 | Out |
| To varying degrees, EPS always occurs after pelvic exenteration if there has been no reconstruction (NEW) |  |  | 7.07 | 56.9 | Out |
| Use of neoadjuvant chemotherapy has an important influence on the development of EPS (NEW) |  |  | 11.2 | 44.6 | Out |
| **Empty Pelvis Syndrome Mitigation Domain Longlisted Statements** |  |  | **Healthcare Professional Voting (%)** | | **Consensus** |
|  |  |  | **Votes 1 – 3** | **Votes 7 – 9** |  |
| Mobilisation of other structures to fill or exclude the empty pelvis from small bowel, i.e., caecum, bladder, uterus, or spare bowel mesentery |  |  | 1.10 | 73.8 | In |
| The use of a bulky myocutaneous flap in order to achieve some pelvic filling |  |  | 2.08 | 89.2 | In |
| The use of a muscle-only flap to fill the pelvis |  |  | 4.65 | 60 | In |
| The use of an omentoplasty for pelvic filling |  |  | 1.08 | 80 | In |
| Use of multiple techniques to ensure the pelvis is adequately filled |  |  | 1.05 | 87.7 | In |
| Using a colorectal anastomosis or coloanal anastomosis to fill the pelvis (NEW) |  |  | 19.4 | 35.4 | Out |
| Using an orthotopic neobladder to fill the pelvis (NEW) |  |  | 27.0 | 15.4 | Out |
| Utilisation of a sigmoid flap to fill the pelvis in cases of partial vaginectomy (short segment of sigmoid brought to the perineal defect, spatulated and used for creation of a neovagina) (NEW) |  |  | 22.6 | 16.9 | Out |

Table S3 – Statements, their corresponding votes from the second Delphi round, and whether they progressed or were dropped. (NEW) – denotes a statement that was generated from thematic analysis of open questions in round one. Statements with * were modified based on thematic analysis of comments from the first Delphi round; EPS – empty pelvis syndrome.
