# Supplementary material for: The empty pelvis syndrome: a core data set from the PelvEx collaborative
Source: Br J Surg. 2024 Mar 8;111(3):znae042. doi: 10.1093/bjs/znae042 (PMC10921833; doi:10.1093/bjs/znae042)
Supplement: znae042_Supplementary_Data [file znae042_supplementary_data.zip › Table_S4.docx]

| **Specific practice on mitigation of the empty pelvis syndrome** | **Responses**  **n (%)** |
| --- | --- |
| **Use of operatively placed drains** | 37/78 (47.4) gave a response on drains |
| **Drain types used:** | 32/37 (86.5) gave a type of drain |
| Robinson | 4/32 (12.5) |
| Jackson-Pratt | 4/32 (12.5) |
| Blake | 6/32 (18.8) |
| Pezzer | 1/32 (3.13) |
| Yeates | 1/32 (3.13) |
| Non-suction drain | 4/32 (12.5) |
| Suction drain | 5/32 (15.6) |
| Unspecified drain | 7/32 (21.9) |
| **Drain positions:** | 21/37 (56.8) specified drain positions |
| Pelvis only | 7/21 (33.3) |
| Perineum only | 3/21 (14.3) |
| Abdomen only | 2/21 (9.52) |
| Urinary diversion only | 1/21 (4.76) |
| Pelvis and perineum | 5/21 (23.8) |
| Abdomen and perineum | 2/21 (9.52) |
| Abdomen and pelvis | 1/21 (4.76) |
| **Drainage duration:** | 6/37 (16.2) specified duration of drainage |
| Just prior to going home | 1/6 (16.7) |
| When drainage minimal | 2/6 (33.3) |
| Two to three days | 1/6 (16.7) |
| At fourteen days with check of drain creatinine | 1/6 (16.7) |
| Until ureteric stents out and CRP <100 | 1/6 (16.7) |
| **Preferences on reconstructive flaps** | 47/78 (60.3) gave a response on flaps |
| Use of a combination of multiple flaps | 5/47 (10.6) |
| Use of different flaps dependent on the circumstances | 11.47 (23.4) |
| **Flaps used:** | A total of 70 flaps were reported |
| Gluteal | 14/70 (20) |
| Rectus | 29/70 (41.4) |
| Omentoplasty | 5/70 (7.14) |
| Gracilis | 9/70 (12.9) |
| Anterolateral thigh flap | 3/70 (4.29) |
| Lotus petal | 1/70 (1.43) |
| Unspecified thigh flap | 2/70 (2.86) |
| Unspecified myocutaneous flap | 7/70 (10) |
| **Use of surgical implants** | 18/78 (23.1) gave a response on implants |
| Use of mesh for rectus flap donor site reinforcement | 3/18 (16.7) |
| **Implants used:** | A total of 16 implants were reported |
| SurgiMend mesh | 3/16 (18.8) |
| Strattice mesh | 1/16 (6.25) |
| OviTex mesh | 1/16 (6.25) |
| Permacol mesh | 2/16 (12.5) |
| Vicryl mesh | 1/16 (6.25) |
| Phasix ST mesh | 1/16 (6.25) |
| Unspecified collagen mesh | 3/16 (18.8) |
| Unspecified bioprosthetic mesh | 2/16 (12.5) |
| Bakri balloon | 1/16 (6.25) |
| Silicone breast implant | 1/16 (6.25) |

Table S4 – Summary of responses to open survey questions in round one on strategies used for reconstruction.
