# Supplementary material for: The empty pelvis syndrome: a core data set from the PelvEx collaborative
Source: Br J Surg. 2024 Mar 8;111(3):znae042. doi: 10.1093/bjs/znae042 (PMC10921833; doi:10.1093/bjs/znae042)
Supplement: znae042_Supplementary_Data [file znae042_supplementary_data.zip › Table_S5.docx]

| **EPS core outcome set domain statements** | **Patient representative votes**  **n (%)** | | **Healthcare professional votes**  **n (%)** | | |
| --- | --- | --- | --- | --- | --- |
|  | Yes | No | Yes | No | Unsure |
| Bowel obstruction | 12 (100) | 0 (0) | 67 (82.7) | 8 (9.88) | 6 (7.4) |
| Enteroperineal fistula | 12 (100) | 0 (0) | 81 (96.4) | 2 (2.38) | 1 (1.19) |
| Quality of life | 12 (100) | 0 (0) | 80 (92) | 6 (87) | 1 (87) |
| Rate of return to theatre and use of interventional radiology to manage EPS complications | 12 (100) | 0 (0) | 78 (88.6) | 4 (4.55) | 6 (6.81) |
| Infected post-operative pelvic fluid collection or abscess | 12 (100) | 0 (0) | 83 (94.3) | 3 (3.41) | 2 (2.27) |
| Post-operative mortality rate | 7 (58.3) | 5 (41.7) | 22 (25.3) | 47 (54.0) | 18 (20.7) |
| Chronic perineal sinus | 12 (100) | 0 (0) | 78 (91.8) | 3 (3.53) | 4 (4.71) |
| Flap related morbidity | 12 (100) | 0 (0) | 39 (63.9) | 14 (23) | 8 (13.1) |
| **EPS pathophysiology domain statements** |  | | **Healthcare professional votes** | | |
|  |  | | **Yes** | **No** | **Unsure** |
| Radiotherapy induced damage contributes to EPS |  | | 76 (86.4) | 10 (11.3) | 4 (4.55) |
| Lack of pelvic filling leads to different complications to those relating to the perineal wound |  | | 76 (86.4) | 1 (1.13) | 11 (12.5) |
| Small bowel falling into the empty pelvic cavity contributes to EPS |  | | 74 (85.1) | 7 (8.05) | 6 (6.90) |
| The greater the magnitude and radicality of PE the worse the complications from EPS |  | | 73 (88.0) | 5 (6.02) | 5 (6.02) |
| EPS is multi-factorial and unpredictable |  | | 73 (88.0) | 6 (7.06) | 6 (7.06) |
| Methods of reconstruction to fill the empty pelvis following PE influences EPS |  | | 71 (80.7) | 5 (5.68) | 12 (13.6) |

Table S5 – Summary of voting at consensus meetings for inclusion into the final core outcome set and pathophysiology domains. Patient representatives did not vote in the pathophysiology domain; EPS – empty pelvis syndrome.
